# Endothelial Dysfunction and Inflammation: Immunity in Rheumatoid Arthritis

**DOI:** 10.1155/2016/6813016

**Published:** 2016-03-30

**Authors:** XueZhi Yang, Yan Chang, Wei Wei

**Affiliations:** Institute of Clinical Pharmacology, Anhui Medical University, Key Laboratory of Anti-Inflammatory and Immune Medicine, Ministry of Education, Hefei 230032, China

## Abstract

Inflammation, as a feature of rheumatoid arthritis (RA), leads to the activation of endothelial cells (ECs). Activated ECs induce atherosclerosis through an increased expression of leukocyte adhesion molecules. Endothelial dysfunction (ED) is recognized as a failure of endothelial repair mechanisms. It is also an early preclinical marker of atherosclerosis and is commonly found in RA patients. RA is now established as an independent cardiovascular risk factor, while mechanistic determinants of ED in RA are still poorly understood. An expanding body of study has shown that EC at a site of RA is both active participant and regulator of inflammatory process. Over the last decade, a role for endothelial dysfunction in RA associated with cardiovascular disease (CVD) has been hypothesized. At the same time, several maintenance drugs targeting this phenomenon have been tested, which has promising results. Assessment of endothelial function may be a useful tool to identify and monitor RA patients.

## 1. Introduction

Cardiovascular disease (CVD) is a serious long-term complication of chronic systemic inflammatory diseases including rheumatoid arthritis (RA) and systemic lupus erythematosus (SLE). RA is characterized by inflammation and the formation of atherosclerosis can be exacerbated and accelerated by systemic inflammation [1, 2]. Compared to patients without RA, cardiovascular morbidity and mortality increase in RA patients. Cardiovascular mortality occurs within 8 years of initial RA symptoms, but the pathophysiological process which proved to exhibit endothelial dysfunction (ED) probably starts much earlier [3–5].

ED is equal to impaired endothelial mechanism, which is first identified in RA patients by Bergholm and colleagues in 2002 [6]. The reason for ED in RA may partly be due to the altered quantity or function of endothelial progenitor cells, which are involved in vasculogenesis and vascular repair [7].

The clinical utility of biomarkers for ED in RA remains unclear. Biomarkers currently under investigation, such as circulating endothelial progenitor cells, may prove to be useful markers of ED. The early development of atherosclerosis and its subsequent progression are closely associated with ED [8]. It may be an appropriate way to use ED as an early indicator of atherosclerosis, thereby reducing risk factor or conducting pharmacologic intervention.

Despite changes in the course of the disease in recent years and new therapeutic options, there is still no evidence that any particular intervention can reduce CVD risk in RA [9]. Thus, drugs targeting ED may be a potential ancillary class in treating RA patients.

## 2. Normal ECs Function

Endothelium is a selectively permeable barrier between vascular wall and bloodstream. In noninflamed tissue, it is responsible for maintaining blood fluidity and regulating blood flow and is able to control vascular wall permeability, quiesce circulating leukocytes [10–14]. Endothelium is also one of the first protective barriers against foreign invasion. These invasions include mechanical stimuli, such as pressure and shear stress, and chemical stimuli, such as hormones and locally secreted vasoactive substances. When endothelium senses stimuli, it releases factors to regulate hemostasis, cell growth, vasomotor function, and inflammatory processes. Among these factors, vasoactive substances play a vital role in inflammation. It is fallen into vasodilator substances and vasoconstrictors. Vasodilator substances include nitric oxide (NO), prostacyclin, and endothelium-derived hyperpolarizing factors. Vasoconstrictors include endothelin-1, angiotensin II (Ang II), and thromboxane A2 [14].

## 3. ECs and Inflammation and Immunity

### 3.1. ECs and Inflammation

 ECs play a major role in the initiation of inflammatory process and have persistent effect on the process. During inflammatory process, the phenotype of ECs turns to be activated [15]. ECs activation is usually classified into two types. Type I activation is a rapid but transient response. It initiates the endothelial interaction with leukocyte and platelets through loosing the ECs junctions and exports Weibel-Palade bodies to release the von Willebrand factor and P-selectin [15]. Type II activation is a slower but more persistent response which invokes the expression of a variety of proinflammatory cytokines and adhesion molecules. The primary mediators of type II activation are tumour necrosis factor-*α* (TNF-*α*) and interleukin-1 (IL-1) derived principally from activated leukocytes [15].

In inflammation, ECs activation induces increased vascular permeability for plasma proteins, the expression of proinflammatory cytokines, chemokines and enzymes, and an upregulation of adhesion molecules [16–18]. Nuclear transcription factor-*κ*B (NF-*κ*B) regulates the expression of adhesion molecules, such as intercellular adhesion molecule-1 (ICAM-1), vascular cell adhesion molecule-1, and E-selectin that play a pivotal role in leukocyte-endothelium interactions. Chemokines, such as monocyte chemoattractant protein 1 and IL-8, contribute to the inflammatory ECs response and are initiated through activation of the classical NF-*κ*B pathway [19].

### 3.2. ECs and Immunity

**Figure 1:**
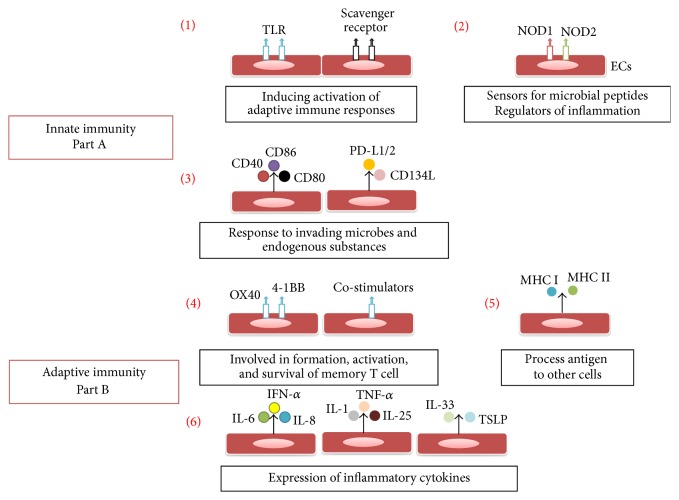
ECs and innate and adaptive immunity. Part A. Innate immunity: (1) ECs express CD36 scavenger receptor and the TLRs, which trigger signals resulting in proinflammatory gene expression, leukocyte chemotaxis, phagocytosis, cytotoxicity, and activation of adaptive immune responses. (2) Receptors NODs 1 and 2 work as sensors for microbial peptides and regulators of inflammation. (3) ECs respond to invading microbes and endogenous substances by producing inflammatory mediators and expressing surface molecules, such as CD40, CD80, CD86, CD134L, PD-L1, and PD-L2. Part B. Adaptive immunity: (4) Activated ECs express costimulators including OX40 ligand and 4-1BB ligand, which are involved in formation, activation, and survival of memory T cell. (5) ECs express MHC I and MHC II class molecules and process antigen. (6) ECs secrete cytokines, such like IL-1, -6, -8, -25, -33, TNF-*α*. TNF*α*, and TSLP, which regulate and shape adaptive and innate immune responses to control the recruitment and influx of immune cells to sites of action.

Endothelium is among the first line of the body's defense system; it encounters and combats the perturbation of invading microbes and endogenous substances in response to tissue damage. ECs respond to these contacts by producing inflammatory mediators and expressing surface molecules, such as CD40, CD80, CD86, CD134L, programmed death-ligand 1 (PD-L1), and PD-L2 [20]. ECs also have been reported to exert their role in immune system via various receptors. Nucleotide-binding oligomerization domains (NODs), cytosolic proteins, are able to sense microbial peptides and regulate inflammation to mediate innate immunity [21]. It is also upregulated in response to lipopolysaccharide (LPS) and proinflammatory cytokines [20]. Specifically, ECs can secrete IL-8 (a proinflammatory cytokine) in a NOD1-dependent manner in response to microbial stimulation [22, 23]. ECs have also been shown to express the NOD2 receptor, which recognizes the bacterial peptidoglycan and muramyl dipeptide [20]. Moreover, ECs express a kind of pattern recognition receptors, such as CD36 scavenger receptor which bind to advanced glycation end-products. ECs also express the toll-like receptors (TLRs). LPS has been shown to modify native low density lipoprotein that can trigger TLR2, TLR4, and TLR6 signaling, thereby mediating the activation of inflammatory cells [24]. For example, LPS-induced ECs can produce IL-1, IL-8, and monocyte chemotactic protein-1 via TLR4 [25–28]. These receptors induce a coordinated signaling network that ultimately activates NF-*κ*B and the subsequent production of chemoattractants, proinflammatory cytokines, and adhesion molecules [21]. TLR3, TLR7, and TLR8 play an important role in detecting viral RNA and activating innate immune responses against viruses, among which TLR3 has been reported expressed by ECs. Interferon-*α* (IFN-*α*) is vital in regulating innate immunity against viruses and inducing TLR3 expression. It has been reported that ECs can also express IFN-*α* [29]. In addition, ECs also express TLR9 that recognizes bacterial and viral DNA [30] (Figure 1).

When innate immunity fails to eliminate inflammatory stimuli, the adaptive immune response will be triggered. This change will turn acute inflammation into chronic inflammation. ECs are involved in chronic inflammation by acting as antigen presenting cells (APCs) and via interactions with specialized effector cells. It has been confirmed that ECs express MHC I and MHC II class molecules and process antigen to other cells* in vitro*. But both of them are still debatable* in vivo* [31]. In addition, activated ECs also express costimulators including OX40 ligand, 4-1BB ligand, and inducible costimulator ligand, which are involved in formation, activation, and survival of memory T cell [32, 33]. Meanwhile, ECs treated with IFN-*γ* effectively induce CD4+ and CD8+ memory T cells to proliferate and produce cytokines [33]. Moreover, ECs also secrete cytokines which regulate and shape adaptive and innate immune responses to control the recruitment and influx of immune cells to sites of action [34]. Among these mediators between immune responses, the central cytokines are IL-25, IL-33, and thymic stromal lymphopoietin (Figure 1).

## 4. Endothelial Dysfunction in RA

The inflammatory processes of RA and CVD are remarkably similar, suggesting that RA disease-related inflammation might contribute to the excess CVD risk [35]. The reason for this is unclear but may be due to ED. ED is a preclinical marker of atherosclerosis and is commonly detected in RA patients. However, the precise pathophysiological mechanisms of ED in RA are still ill defined. Previous study suggested that ED may be related to increased cellular adhesion molecules, accumulation of reactive oxygen species, and changed production such like NO [36–38]. ED leads to a shift of ECs actions by various chemokines, cytokines, and other factors. The shift finally induces reduced vasodilation, proinflammatory state, and proliferative and prothrombotic properties [39]. It is noteworthy that ED may occur differentially in different vascular beds [40]. Evidence suggests that coronary microvascular disease is apparent in the absence of macrovascular disease in RA [41].

### 4.1. Chemokines and ED in RA

The synovium of RA patients has a pink or even red appearance due to the increased number of blood vessels. ECs eventually become vessels through primarily forming tubes together and move towards chemokine gradients through activation of chemokine receptors [42]. Chemokines of the CXC family are key mediators of angiogenesis in both physiological and pathological conditions. CXC chemokines are commonly classified into angiostatic chemokines (inhibiting angiogenesis) and angiogenic chemokines (promoting angiogenesis), based on the presence of a tripeptide ELR (Glu-Leu-Arg) motif preceding the first conserved cysteine residue [43].

**Figure 2:**
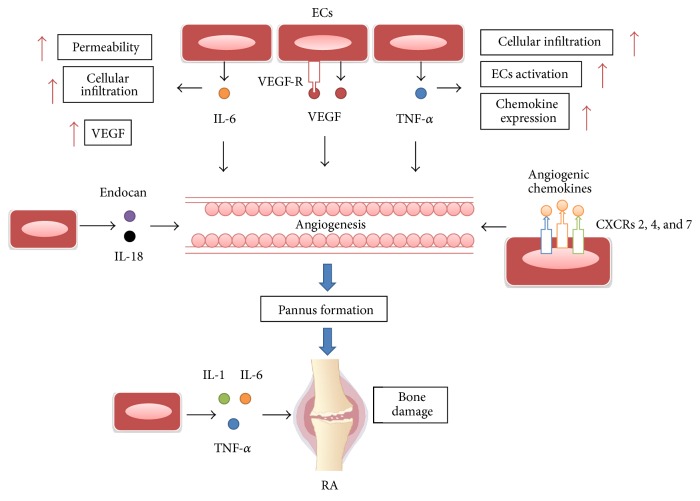
ECs are essential for angiogenesis in RA. VEGF secreted by ECs and other cells inducing angiogenesis by acting on ECs, promoting cell mitogenesis, cell migration, and lumen formation. TNF-*α* can enhance chemokine expression, ECs activation, and angiogenesis. In addition, TNF-*α*, IL-1, and IL-6 can cause bone damage. IL-6 can induce VEGF production in RA FLS and increase permeability and cell infiltration. IL-6, endocan, and IL-18 can also induce angiogenesis. Angiogenic chemokines activate ECs through chemokine receptor CXCR2, CXCR4, and CXCR7 and then induce angiogenesis.

Angiogenic chemokines usually have an ELR motif [44]. In mice and humans, all ELR+ chemokines signal via C-X-C chemokine receptor 2 (CXCR2), but in humans chemokine (C-X-C motif) ligand 6 (CXCL6) and CXCL8 also bind to CXCR1 [45]. These two receptors are expressed by ECs and their ability to induce chemotaxis has been confirmed. However, CXCR2 is generally assumed as one of the major angiogenic receptors also in humans, since some ECs that express CXCR2 only can migrate in response to angiogenic chemokines [46]. CXCR2 has been shown to bind to chemokine CXCL1, CXCL2, CXCL3, CXCL5, CXCL6, CXCL7, and CXCL8 [47]. It has been reported that the inflammation of collagen-induced arthritis (CIA) mice can also be improved through a peptide derived from the angiostatic chemokine CXCL4 [48]. CXCL8 is able to activate human umbilical vein endothelial cells. It has been demonstrated that the angiogenic activity of RA synovial tissue homogenate can be decreased by incubation with anti-CXCL8 antibodies [49]. The exception currently known is CXCL12, which lacks the ELR motif and is expressed in RA fibroblast-like synoviocytes (FLS) [50]. CXCL12, which usually combines with CXCR4 and CXCR7, can inhibit the proangiogenic activity of ELR+ chemokines and vascular endothelial growth factor (VEGF) [50]. As an instance of the thesis that CXCL12 and its receptor CXCR4 are involved in the formation of blood vessels in RA, angiogenic properties of RA synovial fluid were significantly decreased after incubation with anti-CXCL12 antibodies [51]. It has been reported that CIA mice can also be improved through CXCR4 inhibitor [51]. CXCR7 has been reported to fail to support CXCL12 induced human endothelial progenitor cells (EPCs) migration, proliferation, or NO production but it mediated human EPCs survival exclusively [52]. In addition, the expression of CC chemokine ligand 28 and CCR10 is high in RA synovial tissue and can induce EPC migration into RA joints [53] (Figure 2).

Conversely, angiostatic chemokines commonly lack the ELR motif and primarily bind to CXCR3 [54]. The CXC chemokines, CXCL4, CXCL9, CXCL10, CXCL11, CXCL4L1, and the CC chemokine, CCL21, activate CXCR3, a cell-surface G protein-coupled receptor, which can be detected in ECs [47]. The angiostatic functions of these chemokines depend on the capability of CXCR3 to inhibit the proliferation of ECs [55]. CXCL4 is an inhibitor of ECs proliferation and migration either through interaction with CXCR3 or other ways [56, 57]. These effects depend in part on their capability to block the binding of VEGF to its receptors. Unlike CXCL4, CXCL4L1 (nonallelic variant of CXCL4) induces chemokinesis of ECs but does not participate in ECs proliferation [58]. However, the exact effect of these angiostatic chemokines on angiogenesis in RA is still unknown [53] (Figure 2).

### 4.2. Cytokines and ED in RA

It is known that inflammatory cytokines play a pivotal role in driving the disease process in RA. The cytokines synthesized by ECs that have been extensively studied are TNF-*α*, IL-1, and IL-6 [59, 60]. TNF-*α* has been studied for several years for its vital role in joint destruction and its control toward other proinflammatory cytokines. It also increases cellular infiltration in the synovium through enhancing chemokines expression, ECs activation, and angiogenesis [59]. Recent study specifically showed that IL-17 had major procoagulant and prothrombotic effects on vessels when combined with TNF-*α* [61]. TNF-*α* and IL-1 both cause bone damage which is the hallmark of RA pathogenesis [62, 63]. Recent study found that TNF-*α* and IL-1 can increase the expression of GRP78/BiP, a representative ER chaperone which has a high expression in RA synovium and FLS [64]. IL-33, as a member of IL-1 family, is produced when ECs sense stimuli. IL-33 is able to induce angiogenesis and ECs activation [65]. In animal models of RA, IL-1 works with IL-6 during the early phases of disease, acting on ECs to secrete cytokines like IL-8 and monocyte chemotactic protein 1 to attract monocytes. After activation by IL-6, ECs participate in the development of proinflammatory process. The joint destruction and disease progression of RA patients are related to high expression of IL-6/sIL-6R complex in synovial fluids [66]. IL-6 further upregulates chemokines that attract T cells, leading to enhanced cellular infiltration and beginning the transition from an acute inflammatory disease to RA [67]. IL-6 increases the concentration of VEGF in RA FLS to regulate vascular permeability, and increased permeability results in more inflammatory cells recruiting into the tissues and damage exacerbation [68]. In addition, study demonstrated that the absence of IL-6 induces complete protection against arthritis in CIA mice and an anti-mouse IL-6R monoclonal antibody inhibits development of arthritis in CIA mice [69, 70]. IL-18 induces ECs migration and angiogenesis which contribute to sustain and develop the pannus formation. IL-18 works through two ways among which are directly activating ECs and indirectly inducing RA FLS to produce VEGF and angiogenic chemokines [71]. Endocan or endothelial cell specific molecule-1 (ESM-1) is highly expressed in RA synovial tissue. The concentration of ESM-1 is increased by IL-1, TNF-*α*, and VEGF and reduced by IFN-*γ* and IL-4. Study demonstrated that endocan induced by adiponectin can stimulate cell invasion, cell migration, and angiogenesis in arthritic joints [72] (Figure 2).

### 4.3. VEGF and ED in RA

VEGF is able to induce the differentiation of ECs and ECs-driven angiogenesis in RA synovium. The vital molecule that controls inflammation-driven angiogenesis is VEGF-A, a heparin-binding growth factor, inducing angiogenesis by acting on ECs, promoting cell mitogenesis, cell migration, and lumen formation [71–76].

VEGF-A signals induce angiogenesis via vascular endothelial cell receptor-1 (VEGFR-1) and VEGFR-2. Cytokines, such as TNF-*α*, can induce the combination of VEGFR-2 and VEGF-A, thereby resulting in angiogenesis at the site of inflammation [77, 78]. Actually, VEGFR-2 has been recognized as the primary receptor in angiogenic signaling [79].

VEGF level was high in synovium, joint synovial fluid, and serum of patients with RA. Up to now, a variety of cytokines have been shown to contribute to the formation of RA pannus, among which VEGF is a vital cytokine that promotes the formation of pannus by specially acting on the ECs [80]. Clavel et al. demonstrated that serum VEGF level and joint swelling, the disease activity score, the ESR, and the CRP of patients with early RA were positively correlated and proposed that VEGF level may serve as an evaluation index for RA [81]. In addition, VEGF also plays a cooperative role with other factors such as TGF-*β*, FGF, IL-1, and TNF-*α* generated by vessels to promote pannus angiogenesis of RA. Inversely, under the control of inflammatory cytokines in some ways, ECs itself also can secrete VEGF to play a role in inflammation response. Study recently demonstrated for the first time that TNF-*α*-induced B cell activating factor is able to regulate VEGF-induced angiogenesis in RA through c-Fos gene [82].

### 4.4. Ang II and ED in RA

Ang II is a cardiovascular mediator, for its primary role in the regulation of blood fluid homeostasis and blood pressure. As a vasoconstrictor, Ang II also has been implicated in the pathogenesis of RA. It can regulate cellular growth, FLS proliferation, and angiogenesis. Study has reported that Ang II can induce ED, vascular hypertrophy, and hypertensive response in adjuvant arthritis (AA) rat model [83, 84]. It also has a direct influence on the progression of the atherosclerotic process via effects on endothelial function, inflammation, fibrinolytic balance, and plaque stability [85].

Ang II has two major receptor subtypes, the Ang II type 1 receptor (AT1R) and Ang II type 2 receptor (AT2R). AT1R and AT2R, expressed in most organs and tissues, are involved in RA and CVD [86]. For example, it has been previously reported that the AT1R is present and upregulated in the synovium of RA patients [87, 88]. Ang II exerts both its prostress and proinflammatory roles mainly through stimulation of AT1R [89]. In addition, AT1R blockers are known to have direct or indirect anti-inflammatory actions [90]. ED in the circumstance of CVD is partly dependent on the production of reactive oxygen species (ROS). AT1R-induced ROS not only destroys NO but also reduces NO formation in the endothelium [91]. Kawakami et al. have demonstrated that AT1R and AT2R are expressed in articular chondrocytes of RA patients. IL-1 is able to regulate the expression of these two receptors [92]. The AT2 receptor antagonises many effects of the AT1 receptor, for example, cell proliferation. This antagonism includes a direct binding of the AT2 protein to the AT1 receptor. However, it is still debatable in the functions of AT2R; the majority support the concept that the AT2R has antifibrotic, antiproliferative, and anti-inflammatory effects [93, 94].

## 5. Therapeutic Potential of ECs in RA

Increased cardiovascular morbidity and mortality in RA patients have been widely reported. However, to date, drugs treating RA are primarily aimed at prevention of swelling and pain and inhibition of bone erosions. Even CVD risk seems to be higher after taking some drugs. For example, classical nonsteroidal anti-inflammatory drugs and new selective inhibitors of cyclooxygenase-2 have been successfully used for several decades in the treatment of RA patients. However, in recent years, these drugs have been recognized to increase cardiovascular risk. This risk exists and can be explained by a blood pressure increasing effect and the development of ED [95].

Therefore, drugs effectively improve ED in RA patients which may be a potential way used in monotherapy or drug combination as an assist. The only class of medications that specifically target endothelial function is PDE5 inhibitors [96]. But there are still many drugs protecting cardiovascular system in other ways.

Methotrexate (MTX), a gold standard drug in the treatment of RA, has been shown to inhibit angiogenesis and suppresses the expression of E-selectin and ICAM-1 from dermal ECs. Recently, Shaker et al. also demonstrated that MTX cause reduction in the amount of VEGF expression via an antiangiogenesis mechanism [97]. Moreover, other study reported that MTX therapeutically conditions vascular endothelium via activation of AMPK-CREB. This may contribute to MTX against CVD in RA patients [94].

Growing evidence suggests that TNF-*α* inhibitors may prevent CVD in RA patients. For example, etanercept induced a significant decrease in left ventricular mass index (a strong marker of cardiovascular mortality and vessel abnormalities) with medium-term treatment [98]. EPCs promote angiogenesis and vascular repair. The disease activity of circulating EPCs can proportionally reduce by a short-term treatment with TNF-*α* inhibitor. ED also can be affected through suppressing the inflammatory process of RA [99]. These results need to be confirmed by larger studies and with other TNF-*α* inhibitors.

Avastin is a monoclonal antibody for VEGF. Study reported that Avastin can decrease the arthritis index, target-to-nontarget ratio, and synovial pathological injury index of type II collagen-induced arthritis rats [100]. Similarly, bevacizumab, as the first antibody for VEGF, has shown initial preclinical and clinical activity in treating RA [101].

Schulz et al. demonstrated for the first time that, in AA rat model, losartan, an AT1R inhibitor, remarkably reversed the relaxation of ECs-derived hyperpolarizing factor that is damaged. Study also observed that losartan directly inhibited the leukocyte-endothelium interactions [102]. Similarly, AT1R inhibitor, irbesartan, has been demonstrated to be able to improve ED in antigen-induced arthritis [103]. Generally, these findings reveal that AT1R is a potential target to treat ED and may through this way exert its therapeutic effect.

## 6. Concluding Remarks

Evidence has indicated the important role of ED in increased cardiovascular risk in RA patients. On the one hand, drugs targeting ED are of interest from the dual perspectives of disease modification and cardiovascular risk reduction when applied to monotherapy. On the other hand, these drugs may at least act as effective adjunctive therapy in drug combination for disease control in RA patients. However, there are still many gaps in our knowledge regarding these potentially ECs. This is exemplified by the complexity of testing the ECs function in human body. This will still require a uniform approach of experimental studies in humans and nonhuman models. Specifically in patients with RA, it remains to be determined whether simple control of inflammation will be sufficient to restore ECs function.

## Abbreviations

AA: Adjuvant arthritis
AT1R: Angiotensin II type 1 receptor
Ang II: Angiotensin II
CXCR2: C-X-C chemokine receptor 2
CVD: Cardiovascular disease
CXCL6: Chemokine (C-X-C motif) ligand 6
DC: Dendritic cell
ED: Endothelial dysfunction
EPCs: Endothelial progenitor cells
ESM-1: Endothelial cell specific molecule 1
IL-1: Interleukin-1
IFN-*α*: Interferon-*α*
ICAM-1: Intercellular adhesion molecule-1
LPS: Lipopolysaccharide
NF-*κ*B: Nuclear transcription factor-*κ*B
NODs: Nucleotide-binding oligomerization domains
NO: Nitric oxide
PD-L1: Programmed death-ligand 1
RA: Rheumatoid arthritis
ROS: Reactive oxygen species
SLE: Systemic lupus erythematosus
TNF-*α*: Tumour necrosis factor-*α*
TLRs: Toll-like receptors
VEGF: Vascular endothelial growth factor
VEGFR-1: Vascular endothelial cell receptor-1.

## References

[1] Ridker P. M., Hennekens C. H., Buring J. E., Rifai N. (2000). C-reactive protein and other markers of inflammation in the prediction of cardiovascular disease in women. *The New England Journal of Medicine*.

[2] Ku I. A., Imboden J. B., Hsue P. Y., Ganz P. (2009). Rheumatoid arthritis: a model of systemic inflammation driving atherosclerosis. *Circulation Journal*.

[3] Goodson N. J., Wiles N. J., Lunt M., Barrett E. M., Silman A. J., Symmons D. P. M. (2002). Mortality in early inflammatory polyarthritis: cardiovascular mortality is increased in seropositive patients. *Arthritis & Rheumatism*.

[4] Gabriel S. E. (2008). Cardiovascular morbidity and mortality in rheumatoid arthritis. *The American Journal of Medicine*.

[5] Mudau M., Genis A., Lochner A., Strijdom H. (2012). Endothelial dysfunction: the early predictor of atherosclerosis. *Cardiovascular Journal of Africa*.

[6] Bergholm R., Leirisalo-Repo M., Vehkavaara S., Mäkimattila S., Taskinen M.-R., Yki-Järvinen H. (2002). Impaired responsiveness to NO in newly diagnosed patients with rheumatoid arthritis. *Arteriosclerosis, Thrombosis, and Vascular Biology*.

[7] Pompilio G., Capogrossi M. C., Pesce M. (2009). Endothelial progenitor cells and cardiovascular homeostasis: clinical implications. *International Journal of Cardiology*.

[8] Sandoo A., Veldhuijzen van Zanten J. J. C. S., Metsios G. S., Carroll D., Kitas G. D. (2011). Vascular function and morphology in rheumatoid arthritis: a systematic review. *Rheumatology*.

[9] Kramer H. R., Giles J. T. (2011). Cardiovascular disease risk in rheumatoid arthritis: progress, debate, and opportunity. *Arthritis Care & Research*.

[10] Mensah G. A. (2007). Healthy endothelium: the scientific basis for cardiovascular health promotion and chronic disease prevention. *Vascular Pharmacology*.

[11] Busse R., Fleming I. (2006). Vascular endothelium and blood flow. *Handbook of Experimental Pharmacology*.

[12] Minshall R. D., Malik A. B. (2006). Transport across the endothelium: regulation of endothelial permeability. *Handbook of Experimental Pharmacology*.

[13] Bazzoni G., Dejana E. (2004). Endothelial cell-to-cell junctions: molecular organization and role in vascular homeostasis. *Physiological Reviews*.

[14] Ley K., Reutershan J. (2006). Leucocyte-endothelial interactions in health and disease. *Handbook of Experimental Pharmacology*.

[15] Pober J. S., Cotran R. S. (1990). The role of endothelial cells in inflammation. *Transplantation*.

[16] Denk A., Goebeler M., Schmid S. (2001). Activation of NF-*κ*B via the I*κ*B kinase complex is both essential and sufficient for proinflammatory gene expression in primary endothelial cells. *The Journal of Biological Chemistry*.

[17] Oppenheimer-Marks N., Lipsky P. E. (1996). Adhesion molecules as targets for the treatment of autoimmune diseases. *Clinical Immunology and Immunopathology*.

[18] Pober J. S., Sessa W. C. (2007). Evolving functions of endothelial cells in inflammation. *Nature Reviews Immunology*.

[19] Kempe S., Kestler H., Lasar A., Wirth T. (2005). NF-*κ*B controls the global pro-inflammatory response in endothelial cells: evidence for the regulation of a pro-atherogenic program. *Nucleic Acids Research*.

[20] Davey M. P., Martin T. M., Planck S. R., Lee J., Zamora D., Rosenbaum J. T. (2006). Human endothelial cells express NOD2/CARD15 and increase IL-6 secretion in response to muramyl dipeptide. *Microvascular Research*.

[21] Takeuchi O., Akira S. (2010). Pattern recognition receptors and inflammation. *Cell*.

[22] Opitz B., Förster S., Hocke A. C. (2005). Nod1-mediated endothelial cell activation by *Chlamydophila pneumoniae*. *Circulation Research*.

[23] Opitz B., Püschel A., Beermann W. (2006). Listeria monocytogenes activated p38 MAPK and induced IL-8 secretion in a nucleotide-binding oligomerization domain 1-dependent manner in endothelial cells. *Journal of Immunology*.

[24] Weber C., Noels H. (2011). Atherosclerosis: current pathogenesis and therapeutic options. *Nature Medicine*.

[25] Faure E., Equils O., Sieling P. A. (2000). Bacterial lipopolysaccharide activates NF-*κ*B through toll-like receptor 4 (TLR-4) in cultured human dermal endothelial cells. Differential expression of TLR-4 and TLR-2 in endothelial cells. *The Journal of Biological Chemistry*.

[26] Anand A. R., Bradley R., Ganju R. K. (2009). LPS-induced MCP-1 expression in human microvascular endothelial cells is mediated by the tyrosine kinase, Pyk2 via the p38 MAPK/NF-*κ*B-dependent pathway. *Molecular Immunology*.

[27] Anand A. R., Cucchiarini M., Terwilliger E. F., Ganju R. K. (2008). The tyrosine kinase Pyk2 mediates lipopolysaccharide-induced IL-8 expression in human endothelial cells. *Journal of Immunology*.

[28] Marceau F., Grassi J., Frobert Y., Bergeron C., Poubelle P. E. (1992). Effects of experimental conditions on the production of interleukin-1*α* and -1*β* by human endothelial cells cultured in vitro. *International Journal of Immunopharmacology*.

[29] Tissari J., Sirén J., Meri S., Julkunen I., Matikainen S. (2005). IFN-*α* enhances TLR3-mediated antiviral cytokine expression in human endothelial and epithelial cells by up-regulating TLR3 expression. *Journal of Immunology*.

[30] El Kebir D., József L., Pan W., Wang L., Filep J. G. (2009). Bacterial DNA activates endothelial cells and promotes neutrophil adherence through TLR9 signaling. *Journal of Immunology*.

[31] Seledtsov V. I., Goncharov A. G., Seledtsova G. V. (2015). Clinically feasible approaches to potentiating cancer cell-based immunotherapies. *Human Vaccines and Immunotherapeutics*.

[32] Satoh S., Suzuki A., Asari Y. (2002). Glomerular endothelium exhibits enhanced expression of costimulatory adhesion molecules, CD80 and CD86, by warm ischemia/reperfusion injury in rats. *Laboratory Investigation*.

[33] Shiao S. L., McNiff J. M., Pober J. S. (2005). Memory T cells and their costimulators in human allograft injury. *The Journal of Immunology*.

[34] Marelli-Berg F. M., Jarmin S. J. (2004). Antigen presentation by the endothelium: a green light for antigen-specific T cell trafficking?. *Immunology Letters*.

[35] Spasovski D., Latifi A., Osmani B. (2013). Determination of the diagnostic values of asymmetric dimethylarginine as an indicator for evaluation of the endothelial dysfunction in patients with rheumatoid arthritis. *Arthritis*.

[36] Favero G., Paganelli C., Buffoli B., Rodella L. F., Rezzani R. (2014). Endothelium and its alterations in cardiovascular diseases: life style intervention. *BioMed Research International*.

[37] Khan B. V., Harrison D. G., Olbrych M. T., Alexander R. W., Medford R. M. (1996). Nitric oxide regulates vascular cell adhesion molecule 1 gene expression and redox-sensitive transcriptional events in human vascular endothelial cells. *Proceedings of the National Academy of Sciences of the United States of America*.

[38] Stocker R., Keaney J. F. (2004). Role of oxidative modifications in atherosclerosis. *Physiological Reviews*.

[39] Versari D., Daghini E., Virdis A., Ghiadoni L., Taddei S. (2009). Endothelial dysfunction as a target for prevention of cardiovascular disease. *Diabetes Care*.

[40] Hill C. E., Phillips J. K., Sandow S. L. (2001). Heterogeneous control of blood flow amongst different vascular beds. *Medicinal Research Reviews*.

[41] Bocci E. B., Delle Monache F., Cesarotti M., Angrisani C., Gerli R. (2005). Recent views on the pathogenesis of cardiovascular damage associated with rheumatoid arthritis. *Recenti Progressi in Medicina*.

[42] Konisti S., Kiriakidis S., Paleolog E. M. (2012). Hypoxia—a key regulator of angiogenesis and inflammation in rheumatoid arthritis. *Nature Reviews Rheumatology*.

[43] Strieter R. M., Polverini P. J., Kunkel S. L. (1995). The functional role of the ELR motif in CXC chemokine-mediated angiogenesis. *Journal of Biological Chemistry*.

[44] Rudolph E. H., Woods J. M. (2005). Chemokine expression and regulation of angiogenesis in rheumatoid arthritis. *Current Pharmaceutical Design*.

[45] Mantovani A., Sica A., Sozzani S., Allavena P., Vecchi A., Locati M. (2004). The chemokine system in diverse forms of macrophage activation and polarization. *Trends in Immunology*.

[46] Heidemann J., Ogawa H., Dwinell M. B. (2003). Angiogenic effects of interleukin 8 (CXCL8) in human intestinal microvascular endothelial cells are mediated by CXCR2. *The Journal of Biological Chemistry*.

[47] Mehrad B., Keane M. P., Strieter R. M. (2007). Chemokines as mediators of angiogenesis. *Thrombosis and Haemostasis*.

[48] Wooley P. H., Schaefer C., Whalen J. D., Dutcher J. A., Counts D. F. (1997). A peptide sequence from platelet factor 4 (CT-112) is effective in the treatment of type II collagen induced arthritis in mice. *Journal of Rheumatology*.

[49] Koch A. E., Volin M. V., Woods J. M. (2001). Regulation of angiogenesis by the C-X-C chemokines interleukin-8 and epithelial neutrophil activating peptide 78 in the rheumatoid joint. *Arthritis and Rheumatism*.

[50] Li M., Ransohoff R. M. (2009). The roles of chemokine CXCL12 in embryonic and brain tumor angiogenesis. *Seminars in Cancer Biology*.

[51] Pablos J. L., Santiago B., Galindo M. (2003). Synoviocyte-derived CXCL12 is displayed on endothelium and induces angiogenesis in rheumatoid arthritis. *Journal of Immunology*.

[52] Yan X., Cai S., Xiong X. (2012). Chemokine receptor CXCR7 mediates human endothelial progenitor cells survival, angiogenesis, but not proliferation. *Journal of Cellular Biochemistry*.

[53] Chen Z., Kim S.-J., Essani A. B. (2015). Characterising the expression and function of CCL28 and its corresponding receptor, CCR10, in RA pathogenesis. *Annals of the Rheumatic Diseases*.

[54] Cole K. E., Strick C. A., Paradis T. J. (1998). Interferon-inducible T cell alpha chemoattractant (I-TAC): a novel non-ELR CXC chemokine with potent activity on activated T cells through selective high affinity binding to CXCR3. *Journal of Experimental Medicine*.

[55] Lasagni L., Francalanci M., Annunziato F. (2003). An alternatively spliced variant of CXCR3 mediates the inhibition of endothelial cell growth induced by IP-10, Mig, and I-TAC, and acts as functional receptor for platelet factor 4. *The Journal of Experimental Medicine*.

[56] Vandercappellen J., Van Damme J., Struyf S. (2011). The role of the CXC chemokines platelet factor-4 (CXCL4/PF-4) and its variant (CXCL4L1/PF-4var) in inflammation, angiogenesis and cancer. *Cytokine and Growth Factor Reviews*.

[57] De Sutter J., Van de Veire N. R., Struyf S., Philippé J., De Buyzere M., Van Damme J. (2012). PF-4var/CXCL4L1 predicts outcome in stable coronary artery disease patients with preserved left ventricular function. *PLoS ONE*.

[58] Sarabi A., Kramp B. K., Drechsler M. (2011). CXCL4L1 inhibits angiogenesis and induces undirected endothelial cell migration without affecting endothelial cell proliferation and monocyte recruitment. *Journal of Thrombosis and Haemostasis*.

[59] Schmedtje J. F., Ji Y.-S., Liu W.-L., DuBois R. N., Runge M. S. (1997). Hypoxia induces cyclooxygenase-2 via the NF-*κ*B p65 transcription factor in human vascular endothelial cells. *Journal of Biological Chemistry*.

[60] Lotz M. (1995). Interleukin-6: a comprehensive review. *Cancer Treatment and Research*.

[61] Hot A., Lenief V., Miossec P. (2012). Combination of IL-17 and TNF*α* induces a pro-inflammatory, pro-coagulant and pro-thrombotic phenotype in human endothelial cells. *Annals of the Rheumatic Diseases*.

[62] Bertolini D. R., Nedwin G. E., Bringman T. S., Smith D. D., Mundy G. R. (1986). Stimulation of bone resorption and inhibition of bone formation in vitro by human tumour necrosis factors. *Nature*.

[63] Joosten L. A. B., Helsen M. M. A., Saxne T., van De Loo F. A. J., Heinegård D., van Den Berg W. B. (1999). IL-1*αβ* blockade prevents cartilage and bone destruction in murine type II collagen-induced arthritis, whereas TNF-*α* blockade only ameliorates joint inflammation. *Journal of Immunology*.

[64] Yoo S.-A., You S., Yoon H.-J. (2012). A novel pathogenic role of the ER chaperone GRP78/BiP in rheumatoid arthritis. *The Journal of Experimental Medicine*.

[65] Nabe T. (2014). Interleukin (IL)-33: new therapeutic target for atopic diseases. *Journal of Pharmacological Sciences*.

[66] Keul R., Heinrich P. C., Müller-Newen G., Muller K., Woo P. (1998). A possible role for soluble IL-6 receptor in the pathogenesis of systemic onset juvenile chronic arthritis. *Cytokine*.

[67] Ferraccioli G., Bracci-Laudiero L., Alivernini S., Gremese E., Tolusso B., de Benedetti F. (2010). Interleukin-1*β* and interleukin-6 in arthritis animal models: roles in the early phase of transition from acute to chronic inflammation and relevance for human rheumatoid arthritis. *Molecular Medicine*.

[68] Nakahara H., Song J., Sugimoto M. (2003). Anti-interleukin-6 receptor antibody therapy reduces vascular endothelial growth factor production in rheumatoid arthritis. *Arthritis & Rheumatism*.

[69] Alonzi T., Fattori E., Lazzaro D. (1998). Interleukin 6 is required for the development of collagen-induced arthritis. *Journal of Experimental Medicine*.

[70] Takagi N., Mihara M., Moriya Y. (1998). Blockage of interleukin-6 receptor ameliorates joint disease in murine collagen-induced arthritis. *Arthritis and Rheumatism*.

[71] Volin M. V., Koch A. E. (2011). Interleukin-18: a mediator of inflammation and angiogenesis in rheumatoid arthritis. *Journal of Interferon and Cytokine Research*.

[72] Kim K. S., Lee Y.-A., Ji H.-I. (2015). Increased expression of endocan in arthritic synovial tissues: effects of adiponectin on the expression of endocan in fibroblast-like synoviocytes. *Molecular Medicine Reports*.

[73] Ferrara N., Henzel W. J. (1989). Pituitary follicular cells secrete a novel heparin-binding growth factor specific for vascular endothelial cells. *Biochemical and Biophysical Research Communications*.

[74] Shweiki D., Itin A., Soffer D., Keshet E. (1992). Vascular endothelial growth factor induced by hypoxia may mediate hypoxia-initiated angiogenesis. *Nature*.

[75] Kampen K. R. (2012). The mechanisms that regulate the localization and overexpression of VEGF receptor-2 are promising therapeutic targets in cancer biology. *Anti-Cancer Drugs*.

[76] Detmar M., Brown L. F., Claffey K. P. (1994). Overexpression of vascular permeability factor/vascular endothelial growth factor and its receptors in psoriasis. *Journal of Experimental Medicine*.

[77] Huggenberger R., Detmar M. (2011). The cutaneous vascular system in chronic skin inflammation. *Journal of Investigative Dermatology Symposium Proceedings*.

[78] Hoeben A., Landuyt B., Highley M. S., Wildiers H., Van Oosterom A. T., De Bruijn E. A. (2004). Vascular endothelial growth factor and angiogenesis. *Pharmacological Reviews*.

[79] Maharaj A. S. R., Saint-Geniez M., Maldonado A. E., D'Amore P. A. (2006). Vascular endothelial growth factor localization in the adult. *The American Journal of Pathology*.

[80] Kim W.-U., Yoo S.-A., Kwok S.-K. (2008). Proinflammatory role of vascular endothelial growth factor in the pathogenesis of rheumatoid arthritis: prospects for therapeutic intervention. *Mediators of Inflammation*.

[81] Clavel G., Bessis N., Lemeiter D. (2007). Angiogenesis markers (VEGF, soluble receptor of VEGF and angiopoietin-1) in very early arthritis and their association with inflammation and joint destruction. *Clinical Immunology*.

[82] Lee G.-H., Lee J., Lee J.-W., Choi W. S., Moon E.-Y. (2013). B cell activating factor-dependent expression of vascular endothelial growth factor in MH7A human synoviocytes stimulated with tumor necrosis factor-*α*. *International Immunopharmacology*.

[83] Suzuki Y., Ruiz-Ortega M., Lorenzo O., Ruperez M., Esteban V., Egido J. (2003). Inflammation and angiotensin II. *International Journal of Biochemistry and Cell Biology*.

[84] Sakuta T., Morita Y., Satoh M., Fox D. A., Kashihara N. (2010). Involvement of the renin–angiotensin system in the development of vascular damage in a rat model of arthritis: effect of angiotensin receptor blockers. *Arthritis & Rheumatism*.

[85] Husain K., Hernandez W., Ansari R. A., Ferder L. (2015). Inflammation, oxidative stress and renin angiotensin system in atherosclerosis. *World Journal of Biological Chemistry*.

[86] Mackenzie A., Dunning L., Ferrell W. R., Lockhart J. C. (2013). Angiotensin II type 1 receptor blockade protects endothelium-derived hyperpolarising factor-mediated relaxation in a rat model of monoarthritis. *Life Sciences*.

[87] Walsh D. A., Suzuki T., Knock G. A., Blake D. R., Polak J. M., Wharton J. (1994). AT1 receptor characteristics of angiotensin analogue binding in human synovium. *British Journal of Pharmacology*.

[88] Price A., Lockhart J. C., Ferrell W. R., Gsell W., McLean S., Sturrock R. D. (2007). Angiotensin II type 1 receptor as a novel therapeutic target in rheumatoid arthritis: In vivo analyses in rodent models of arthritis and ex vivo analyses in human inflammatory synovitis. *Arthritis and Rheumatism*.

[89] Marchesi C., Paradis P., Schiffrin E. L. (2008). Role of the renin-angiotensin system in vascular inflammation. *Trends in Pharmacological Sciences*.

[90] Dagenais N. J., Jamali F. (2005). Protective effects of angiotensin II interruption: evidence for antiinflammatory actions. *Pharmacotherapy*.

[91] Walsh D. A., Catravas J., Wharton J. (2000). Angiotensin converting enzyme in human synovium: increased stromal [125I]351A binding in rheumatoid arthritis. *Annals of the Rheumatic Diseases*.

[92] Kawakami Y., Matsuo K., Murata M. (2012). Expression of angiotensin II receptor-1 in human articular chondrocytes. *Arthritis*.

[93] Steckelings U. M., Kaschina E., Unger T. (2005). The AT2 receptor—a matter of love and hate. *Peptides*.

[94] Thornton C. C., Al-Rashed F., Calay D., Thornton C. C., Al-Rashed F., Calay D. (2015). Methotrexate-mediated activation of an AMPKCREB- dependent pathway: a novel mechanism for vascular protection in chronic systemic inflammation. *Annals of the Rheumatic Diseases*.

[95] Hermann M. (2012). Cardiovascular risk of non-steroidal anti-inflammatory drugs. *Praxis*.

[96] Peixoto C. A., Gomes F. O. D. S. (2015). The role of phosphodiesterase-5 inhibitors in prostatic inflammation: a review. *Journal of Inflammation*.

[97] Shaker O. G., Khairallah M., Rasheed H. M. (2013). Antiangiogenic effect of methotrexate and PUVA on psoriasis. *Cell Biochemistry and Biophysics*.

[98] He M. F., Gao X. P., Li S. C. (2014). Anti-angiogenic effec to fauranofin on HUVECs in vitro and zebrafish in vivo. *European Journal of Pharmacology*.

[99] Spinelli F. R., Metere A., Barbati C. (2013). Effect of therapeutic inhibition of TNF on circulating endothelial progenitor cells in patients with rheumatoid arthritis. *Mediators of Inflammation*.

[100] Wang Y., Da G., Li H., Zheng Y. (2013). Avastin exhibits therapeutic effects on collagen-induced arthritis in rat model. *Inflammation*.

[101] Salesi N., Bossone G., Veltri E. (2005). Clinical experience with bevacizumab in colorectal cancer. *Anticancer Research*.

[102] Schulz E., Jansen T., Wenzel P., Daiber A., Münzel T. (2008). Nitric oxide, tetrahydrobiopterin, oxidative stress, and endothelial dysfunction in hypertension. *Antioxidants and Redox Signaling*.

[103] Silveira K. D., Coelho F. M., Vieira A. T. (2013). Mechanisms of the anti-inflammatory actions of the angiotensin type1 receptor antagonist losartan in experimental models of arthritis. *Peptides*.

